# Expression and Clinical Significance of the Autophagy Proteins BECLIN 1 and LC3 in Ovarian Cancer

**DOI:** 10.1155/2014/462658

**Published:** 2014-07-17

**Authors:** Guido Valente, Federica Morani, Giuseppina Nicotra, Nicola Fusco, Claudia Peracchio, Rossella Titone, Oscar Alabiso, Riccardo Arisio, Dyonissios Katsaros, Chiara Benedetto, Ciro Isidoro

**Affiliations:** ^1^Laboratory of Anatomy Pathology, Department of Translational Medicine, Università del Piemonte Orientale “A. Avogadro”, Via Solaroli 17, 28100 Novara, Italy; ^2^Laboratory of Molecular Pathology and Nanobioimaging, Department of Health Sciences, Università del Piemonte Orientale “A. Avogadro”, Via Solaroli 17, 28100 Novara, Italy; ^3^Unit of Oncology, Department of Translational Medicine, Università del Piemonte Orientale “A. Avogadro”, Via Solaroli 17, 28100 Novara, Italy; ^4^A.O.U. Città della Salute e della Scienza di Torino Presidio O.I.R.M-Sant'Anna Hospital, Corso Spezia No. 60, 10126 Torino, Italy; ^5^Gynaecology and Obstetrics, Department of Surgical Sciences, Sant'Anna Hospital, University of Torino, Corso Spezia No. 60, 10126 Torino, Italy

## Abstract

Autophagy is dysregulated in cancer and might be involved in ovarian carcinogenesis. BECLIN-1, a protein that interacts with either BCL-2 or PI3k class III, plays a critical role in the regulation of both autophagy and cell death. Induction of autophagy is associated with the presence of vacuoles characteristically labelled with the protein LC3. We have studied the biological and clinical significance of BECLIN 1 and LC3 in ovary tumours of different histological types. The positive expression of BECLIN 1 was well correlated with the presence of LC3-positive autophagic vacuoles and was inversely correlated with the expression of BCL-2. The latter inhibits the autophagy function of BECLIN 1. We found that type I tumours, which are less aggressive than type II, were more frequently expressing high level of BECLIN 1. Of note, tumours of histologic grade III expressed low level of BECLIN 1. Consistently, high level of expression of BECLIN 1 and LC3 in tumours is well correlated with the overall survival of the patients. The present data are compatible with the hypotheses that a low level of autophagy favours cancer progression and that ovary cancer with upregulated autophagy has a less aggressive behaviour and is more responsive to chemotherapy.

## 1. Introduction

Epithelial ovary cancers (EOCs) represent the vast majority (approximately 90%) of all ovary tumours. Based on morphological criteria, EOCs are classified as serous (of low and high grade), clear cell, endometrioid, mucinous transitional (Brenner type), mixed mesodermal, and undifferentiated histologic subtypes [1]. The histogenesis of EOC is still debated. Very recently, the traditional view that EOCs arise from the metaplastic transformation of the mesothelium overlying the ovaries has been challenged by a new paradigm suggesting that these carcinomas indeed arise in extraovarian sites and involve the ovaries secondarily [1]. Based on genetic and clinical features, ovarian carcinomas are classified as type I that comprise the low-grade serous, low-grade endometrioid, clear cell, mucinous, and transitional (Brenner) histologic types and as type II that comprise the high-grade serous, high-grade endometrioid, undifferentiated, and mixed mesodermal histologic types [1]. Type I ovarian carcinomas are genetically more stable and clinically indolent and less aggressive than type II ovarian carcinomas [1].

Ovarian cancer ranks as the sixth to eighth most frequent cancer in developed countries [2] and, in spite of the recent progresses made in understanding the genetic and biologic bases [3, 4], it remains the most lethal among all the gynaecologic malignancies, with a 5-year survival of less than 30% [5]. Bad prognosis is essentially due to the fact that diagnosis of ovarian cancers often occurs at a late stage (because of the lack of precocious alarming symptoms) and also due to the recurrence of chemoresistant tumours. Therefore, new biomarkers for early detection and for monitoring the progression of ovarian cancers [6], as well as new therapeutic strategies that could specifically target the chemoresistant clones [3, 4], are needed.

Autophagy, a lysosomal-dependent pathway for the degradation of redundant or damaged cell components, has recently been suggested to play a role in ovarian carcinogenesis and to be a potential therapeutic target to combat this cancer [7]. Autophagy begins with the production of double-membrane vacuoles (named autophagosomes) that entrap the material to be degraded and eventually fuse with lysosomes (reviewed in [7]). The autophagosomes are characteristically marked by the presence of protein LC3 (deriving from posttranslational modifications of a microtubule-associated protein precursor) on their membranes [8]. Among the many proteins that directly or indirectly regulate the autophagy process, BECLIN 1 seems to be of particular relevance in ovarian carcinogenesis. BECLIN 1 was initially isolated as an interactor of the oncogenic antiapoptotic protein BCL-2, and it was reported to be deleted in up to 75% of human ovarian cancers [9, 10]. The monoallelic deletion of BECLIN 1 in mice caused the spontaneous development of tumours, including ovarian cancer, in association with reduced autophagy [11].

To trigger autophagy, BECLIN 1 must release BCL-2 and form dimers which interact with PI3-kinase class III (or Vps34), thus forming an oligomeric complex that can be evidenced by immunohistochemistry or immunofluorescence as definite spots in the cytoplasm [12, 13]. Autophagy-active BECLIN 1 has been proposed as a potential prognostic biomarker in several tumours [13–15]. However, the prognostic significance of BECLIN 1 expression in ovarian carcinomas appears controversial. Shen et al. [16] found that BECLIN 1 expression was significantly higher in benign and borderline ovarian tumours than in malignant EOC, which was consistent with the view that a decreased capacity of autophagy could favour tumorigenesis in the ovary. Recently, this same group confirmed this observation in a larger cohort of patients and also found that low expression of BECLIN 1 and high level of expression of BCL-2 were associated with advanced clinical stage at diagnosis and poor prognosis [17]. In contrast, another study found that BECLIN 1 expression was increased in malignant versus benign ovary tissues and that such high expression was associated with worse prognosis [18]. Increased expression of BECLIN 1 was found also to be associated with the most aggressive endometrioid adenocarcinomas and poor 5-year overall survival, probably because of concomitant tumour hypoxia [19]. In this same line, it was reported that the high expression of LC3A, the marker of autophagosomes, was associated with hypoxia and poor prognosis in clear cell, but not other examined subtypes, ovarian cancers [20].

In this work, we assessed by immunohistochemistry and immunofluorescence the expression of BECLIN 1 and of LC3 in various histologic subtypes of ovarian cancer. The ratio of BECLIN 1 and BCL-2 expression was also determined by western blotting in some selected cases. We noted that type I ovarian carcinomas that are clinically less aggressive than type II were more frequently expressing high level of BECLIN 1. Conversely, low level of BECLIN 1 expression correlated with histologic grade III tumours. No statistically significant association with patient survival was found in the cases judged negative for BECLIN 1 expression. On the other hand, granular-like positivity of BECLIN 1 and LC3, which is indicative of ongoing autophagy, was more frequently observed in tumours from patients with a better survival. These data suggest that ovarian cancer progression is facilitated by low level of intrinsic autophagy and that ovarian cancers with upregulated autophagy are more likely to respond to therapeutic treatments and to progress more slowly.

## 2. Materials and Methods

### 2.1. Patients, Therapeutic Treatments, and Tissue Collection

The present retrospective study includes 61 cases of ovarian carcinomas selected in the years 1999–2004 from the archived materials of the Department of Gynecology of Università di Torino (Italy). All cases were classified according to the current WHO Classification of Neoplasms. All patients underwent surgery. With the exception of those staged as pT1/G1 (for whom no further treatment was required), all patients were thereafter subjected to a standard chemotherapy regimen which included Carboplatin AUC 5/6 and Paclitaxel 175 mg/mq every 3 weeks for 6 cycles, outside of clinical trials. Follow-up ended in 2009. Biopsies were obtained at the time of the first surgery. Formalin-fixed paraffin-embedded tissue sections were prepared and used for diagnostic purposes and for the present investigation. No oral or written informed consent was obtained from the patients for the use of these retrospective samples, since it was not deemed necessary by the local ethics committee. All samples were treated anonymously.

### 2.2. Tissue Immunoreactivity for BECLIN 1, LC3, and BCL-2

Immunohistochemistry and immunofluorescence were performed in deparaffinized tissue sections following our published protocol [13]. Proteins of interest were revealed by subsequent incubation of the tissue with a primary (first step) and a secondary (second step) antibody. In the first step the following primary antibodies, either alone or in appropriate combination, were used: (a) anti-BECLIN 1 mouse monoclonal (BD Pharmingen, San Diego, CA; dilution 1 : 100) or anti-BECLIN 1 rabbit polyclonal (Santa Cruz Biotechnology, Santa Cruz, CA; dilution 1 : 100); (b) anti-LC3 rabbit polyclonal (Novus Biological, Littleton, Colorado; dilution 1 : 500); (c) anti-BCL-2 mouse monoclonal (Santa Cruz Biotechnology, Santa Cruz, CA; dilution 1 : 100). Appropriate secondary antibodies, goat-anti-mouse IgG or goat-anti-rabbit IgG (Sigma-Aldrich Inc., St. Louis, MO; dilution 1 : 200), labelled with horse-radish-peroxidase (for immunohistochemistry) or with FITC or Texas Red fluorescent dye (for immunofluorescence), were used in the second step. The section subjected to immunofluorescence were also stained with the fluorescent dye 4-6-diamidino-2-phenylindole-dihydrochloride (DAPI, 1 : 500 from a stock solution 20 mg/mL; 1 h) to evidence the nucleus. The sections were mounted with Slow-FAD (Light AntiFADE kit, Molecular Probes Invitrogen, Carlsband, CA, USA), observed under a fluorescence microscope (Leica DM1600, Leica Microsystem, Heidelberg, Germany) and representative areas were imaged with a digital camera.

### 2.3. Evaluation of Tissue Positivity for Autophagy Proteins

To assess the immunoreactivity for BECLIN 1 and for LC3 proteins, at least five fields randomly chosen (approximately 5000 cells) per section were evaluated independently by three investigators (GV and CI for IHC; GN and CI for IF). The sample was considered positive only when the immunoreaction presented with a granular-like pattern. For this purpose, high magnification images were used. Only neoplastic cells were counted. The proportion of positive cells over the total number of cells present in the imaged areas were expressed as percentage. A final hybrid score (H) was assigned to each sample, based on the product of a 0–3 scale of staining intensity and of the percentage of positive cells (0–100%), with a possible range of results from 0 to 300. Each biopsy was tested at least two times.

### 2.4. Tissue Western Blotting of BECLIN 1 and of BCL-2

For some samples a frozen biopsy was also available and used for western blotting detection of BECLIN 1 and of BCL-2, following our published protocol [13]. Essentially, a piece of frozen biopsy was homogenized by several cycles of freeze-thawing and sonication in a phosphate buffer containing detergents and protease inhibitors. A 30 *μ*g of protein homogenate was resolved by SDS-polyacrylamide gel electrophoresis and thereafter electrotransferred into a nitrocellulose membrane. Standard procedure for western blotting was used [21] to detect BECLIN 1 and BCL-2, respectively, with a monoclonal antibody (BD Pharmingen; dilution 1 : 250) and a rabbit polyclonal antiserum (Santa Cruz Biotechnology; dilution 1 : 100). After stripping, the filter was reprobed to detect actin, used as a loading marker. Appropriate peroxidase-conjugated secondary antibodies (purchased from Sigma-Aldrich; dilution 1 : 20.000) were used to reveal the immunocomplexes through peroxidase-induced chemiluminescence reaction (Biorad, Hercules, CA, USA).

### 2.5. Statistical Analysis

BECLIN 1 and LC3 granular-like positivity as assessed by IHC and/or IF was correlated to the clinical outcome referred to as complete remission (CR) and overall survival (OS) at 5 years. The odds ratio, the relative risk, and the Chi-square were calculated using the Microsoft Excel XLStat 2010 software. The Fisher's exact test was also employed for pairwise comparison of distributions of categorized groups. A *P* value lower than 0.05 was taken to indicate data statistically significant.

## 3. Results

### 3.1. Histologic Type and Main Clinical Characteristics of Ovary Carcinomas Included in the Study

This retrospective study included 61 cases of ovary carcinomas of various histologic types selected from our archived materials. The tumours were grouped as type I and type II [1]. All patients were subjected to surgery and chemotherapy, following standard criteria based on clinical stage and patient performance status. The following information was available: clinical stage at first diagnosis, histologic type, objective response to chemotherapy, and clinical outcome. Response to therapy regimen was evaluated according to the international guidelines. Clinical outcomes were classified as complete remission (CR) or alternatively as not evidence of disease (NED), that is, disappearance of any evidence of disease during the follow-up or for at least four years), partial remission (PR, ≥ 50% decrease of tumour lesions for at least 24 months), and DOD (dead of disease). Overall survival (OS) was calculated from the time of first diagnosis to the end of the follow-up, which terminated in 2009. The database with the histologic, clinical, and patients' main information of the cases included in the present study is reported in Supplementary Table ST1 (see Supplementary Table ST1 in Supplementary Material available online at http://dx.doi.org/10.1155/2014/462658).

### 3.2. Immunodetection of BECLIN 1 in Ovary Cancer Tissues

**Figure 1:**
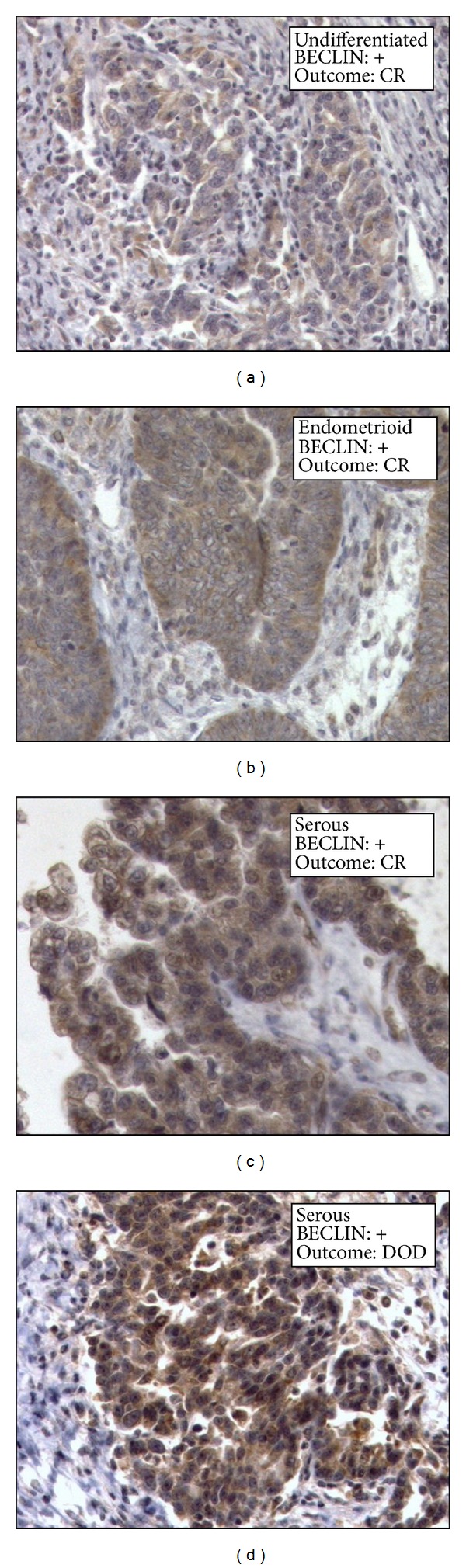
Immunohistochemical detection of BECLIN 1. Selection of representative cases. The histologic type and the clinical outcome (CR: complete remission; DOD: dead of disease) are indicated. Magnification 220x.

**Figure 2:**
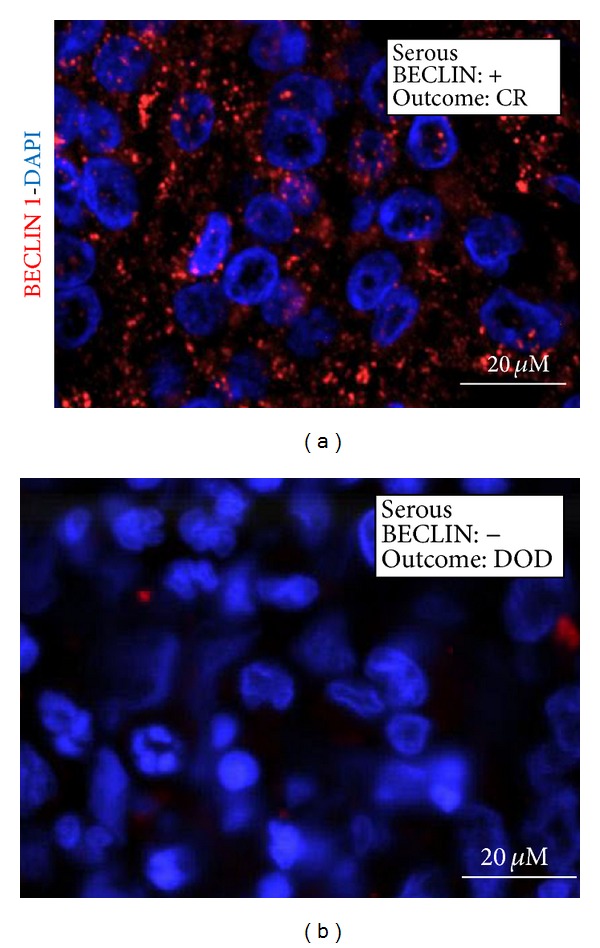
Immunofluorescence detection of BECLIN 1. Selection of representative cases. The histologic type and the clinical outcome (CR: complete remission; DOD: dead of disease) are indicated. The nuclei are evidenced by DAPI staining.

The presence and the cytoplasmic distribution of BECLIN 1 were first analysed by immunohistochemistry (IHC) in paraffin-embedded tissue sections of ovary carcinomas. BECLIN 1 immunoreactivity in tumour cells presented as a faintly detectable staining diffused in the cytoplasm or as discrete stained puncta (referred to as granular-type) clearly evident in the vicinity of the nucleus. The former immunoreactivity pattern was considered as negative, whereas the latter was considered as positive in terms of BECLIN 1 macroaggregates and indicative of active autophagy. A parallel analysis of BECLIN 1 expression was conducted by immunofluorescence (IF) in the same sections. Results from both techniques overlapped, though some cases judged negative on IHC appeared faintly positive on IF, owing to the highest sensitivity of the latter technique. Representative images of BECLIN 1 expression and cellular distribution, as assessed by IHC and IF in selected cases, are shown in Figures 1 and 2, respectively.

### 3.3. Correlation of BECLIN 1 Expression with Histologic Type

Based on the proportion of BECLIN 1-positive cells within the tumour tissue, the samples were initially stratified into four ranges of positivity: <10%; 10–20%; 20–40%; >40%. Based on the intensity (on a 0 to 3 scale) and on the proportion of the cells positive for BECLIN 1 as assessed by IHC, hybrid score (H) was assigned to each section independently by two pathologists (GV and CI). To indicate positivity for BECLIN 1 expression the final threshold was set at ≥20% of cells showing a granular-like staining of intensity ≥2 (H ≥ 40). A high proportion of BECLIN 1-positive cells was reported in 41 out of 61 tumours. Of note, while type II tumours showed an approximately equal distribution of BECLIN 1 positivity (11 positive and 8 negative), as many as 20 out of 23 type I tumours were found highly expressing BECLIN 1. More in detail, >20% BECLIN 1-positive cells (>40 H) were found in the majority of endometrioid adenocarcinomas (11/13) and of serous cystadenocarcinomas (19/27). However, there was no statistically significant association between the extent of BECLIN 1-positive cells and a particular histologic type of ovarian cancer (Table 1).

### 3.4. BECLIN 1 Expression Correlates with Histologic Grading but Not with Pathological Staging at Diagnosis

Next, we looked for any correlation between the extent of BECLIN 1 expression and the aggressiveness of ovarian cancers as mirrored by the histologic grading and the pathological stage at diagnosis. It was found that while tumours with a high expression of BECLIN 1 were equally distributed in I-II and III grade, tumours negative or low expressing BECLIN 1 more frequently (18 out of 20) belonged to grade III (Table 2(a)). This correlation was statistically significant (*P* = 0.004). With regard to the pathological staging, it was found that of the 20 carcinomas with <20% of BECLIN 1-positive cells, 10 were classified as I-II stage and 10 as III-IV stage; of the 41 carcinomas with ≥20% BECLIN 1-positive cells, 24 were of I-II stage and 17 of III-IV stage (Table 2(b)). No significant correlation was found between the positivity for BECLIN 1 and the pathological stage (*P* = 0.7). On the whole, these findings indicate that the absence of BECLIN 1 expression, which likely determines defective autophagy, favours a more malignant phenotype of the tumour, though other factors, independent of the intrinsic autophagy capacity, influence the evolution of the disease and the accompanying general symptoms that lead to the first diagnosis.

### 3.5. Ovarian Carcinomas Highly Expressing BECLIN 1 Associate with Better Patient's Clinical Outcome

We asked about the clinical significance of BECLIN 1 expression in terms of the impact on the posttherapy outcome. The patients were all subjected to surgical removal of the ovaries and annexes, followed by a standard chemotherapeutic treatment protocol. Chemotherapeutics included Carboplatin and Paclitaxel. For seven patients, staged as pT1 and bearing a G1 tumour, no adjuvant chemotherapy was administered. We first correlated the expression of BECLIN 1 with the patient's overall survival (OS) at 5 years. Patients bearing a tumour with a low expression of BECLIN 1 (H < 40) showed no differences in terms of OS, with 9 being dead and 11 still alive at the time of the end of the follow-up (Table 3(a)). By contrast, a statistically significant correlation was found between the high expression of BECLIN 1 (i.e., tumours with ≥20% of positive cells) and patient's OS. In particular, of the 41 patients bearing a tumour highly expressing BECLIN 1, 34 (~83%) were still alive at the end-point of the study and only 7 (17%) died during the observation period. These correlations were statistically significant (*P* < 0.03). We then considered the clinical outcome separately as CR (or NED), PR, and DOD to see any correlation with the expression of BECLIN 1. Amongst the 61 cases, 32 patients (52%) underwent CR. Of these, as many as 24 (75%) were bearing an ovary cancer with ≥20% BECLIN 1-positive cells. Conversely, only 8 out of 32 (25%) patients in CR were bearing a cancer with a ≤20% of BECLIN 1-positive cells (Table 3(b)). PR was more frequently observed in the group of patients bearing a cancer with a high proportion of BECLIN 1-positive tumour cells than in the group of patients bearing a BECLIN-negative cancer (25% versus 15%), and DOD was also less frequent in the former than in the latter group of patients (17% versus 45%). These correlations were, however, not statistically significant (*P* < 0.06). Altogether, these observations support the content that the high expression of BECLIN 1 in ovarian carcinomas associates with a better prognosis. However, no correlation with the clinical outcome was found in the group of patients bearing a tumour negative or low expressing BECLIN 1. We have also performed the analysis of the overall survival probability of the patients by the Kaplan-Meier method (Supplementary Figure  1, SF1). Log-rank test indicated that the association of high expression of BECLIN 1 in the tumour with a good prognosis was statistically significant. Yet, a larger number of patients should be studied in order to substantiate the above finding.

### 3.6. BECLIN 1 and LC3 Double Positivity Predicts a Favourable Prognosis in Ovarian Cancer

**Figure 3:**
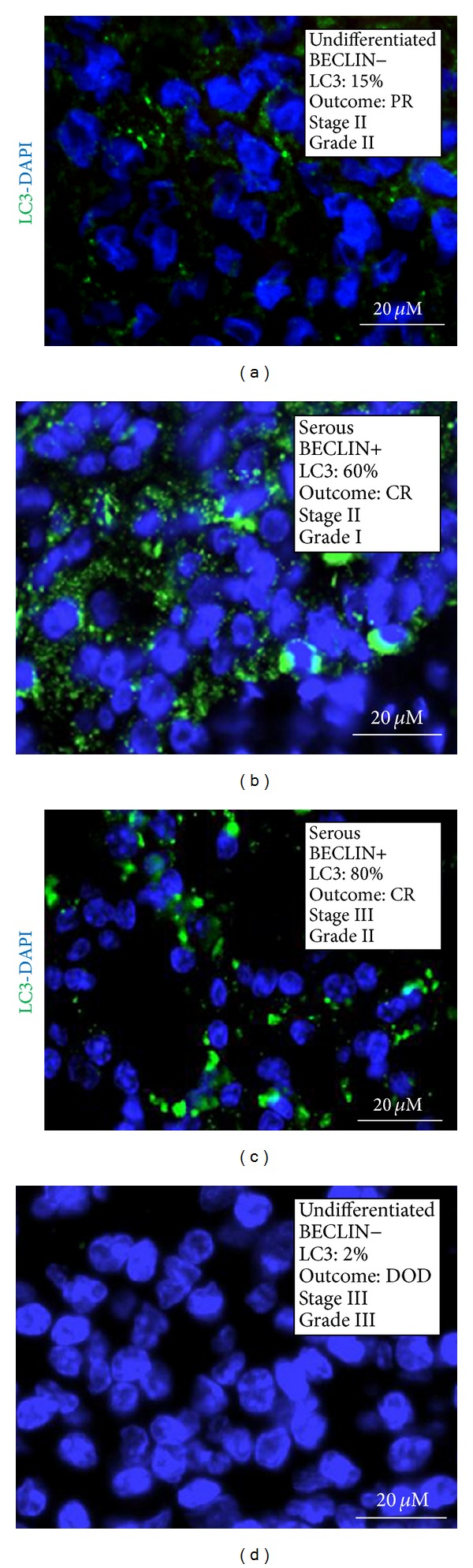
Immunofluorescence staining of LC3 in ovarian cancer tissue sections. Selection of representative cases. The histologic type, the positivity for BECLIN 1 aggregates, the percentage of cells positive for vacuolar LC3, the clinical outcome (CR: complete remission; PR: partial remission; DOD: dead of disease), the pathological stage, and the histologic grade are reported. The nuclei are evidenced by DAPI staining.

In autophagy active cells, the microtubule-associated LC3 protein is posttranslationally translocated into the membranes of autophagosomes [8]. Therefore, the detection of a granular-like staining of LC3 can be assumed* bona fide* as the proof of the presence of autophagic vacuoles (either autophagosomes or autophagolysosomes) in the cell. We analysed by immunofluorescence the expression of LC3 in selected BECLIN 1-positive (*n* = 30) and BECLIN 1-negative cases (*n* = 12). Cells were considered positive for ongoing autophagy when showing a granular-like staining for LC3 and the tumour was considered autophagy-active when ≥20% of the cells were LC3 positive. Examples of LC3 staining in BECLIN 1-positive tumour cells are shown in Figure 3. With a few exceptions, cases judged positive for BECLIN 1 were highly positive also for LC3. On the whole, we found a concordance of 70% between the expression of both BECLIN 1 and LC3.

To further substantiate the involvement of autophagy in the progression and chemotherapeutic response of ovarian carcinomas, we correlated the expression of LC3 with the clinical outcome. When restricted to the group of BECLIN 1-positive tumours, it was found that 20 out of 21 patients bearing a tumour also positive for LC3 were still alive, while 6 out of 9 of those patients bearing a tumour negative for LC3 were DOD, at 5 years after diagnosis (Table 4(a)). These correlations were statistically significant (*P* < 0.0002). Statistics was then applied to the whole group of tumours analyzed for LC3 positivity, including both the BECLIN 1 positive and BECLIN 1 negative. On the whole, 23 out of 24 patients with an LC3-positive tumour were still alive, while 11 out 18 patients with an LC3-negative tumour were DOD, at 5 years after diagnosis (Table 4(b)). Of note, in this case the correlations were even more significant (*P* < 0.0002).

### 3.7. Coexpression of BECLIN 1 and BCL-2 in relation to Autophagy in Ovarian Cancers

**Figure 4:**
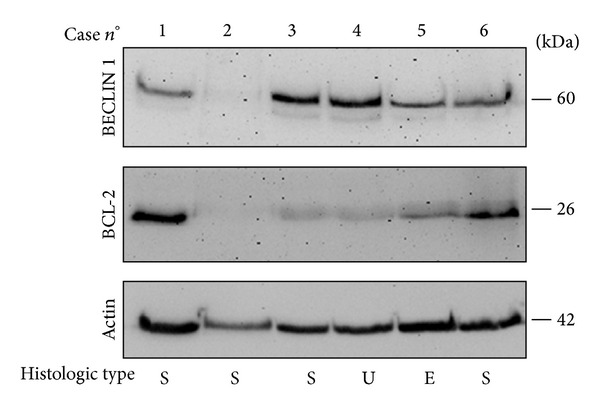
Western blotting analysis of the expression of BECLIN 1 and of BCL-2 proteins in ovarian carcinomas. Selection of representative cases. Tissue homogenates were subsequently probed for BECLIN 1, BCL-2, and actin (the latter was used as reference of homogenate protein loading). The molecular weight of proteins detected with the specific antibodies is indicated. Histologic type: S: serous; U: undifferentiated; E: endometrioid.

**Figure 5:**
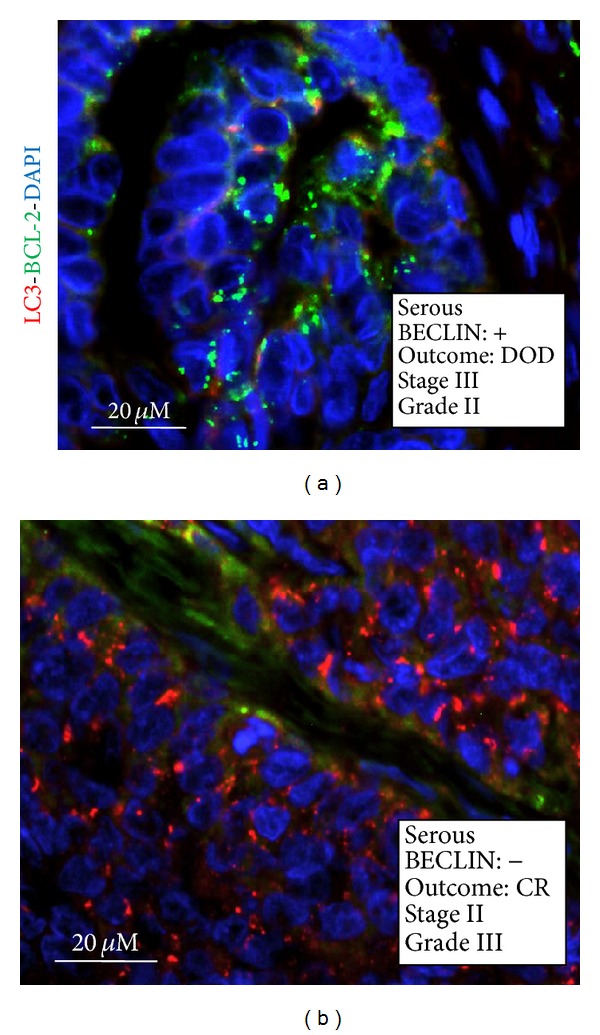
Immunofluorescence staining of LC3 and BCL-2. Selection of representative cases. The histologic type, the positivity for BECLIN 1 aggregates, the clinical outcome (CR: complete remission; DOD: dead of disease), the pathological stage, and the histologic grade are reported. The nuclei are evidenced by DAPI staining.

The interaction of BECLIN 1 with BCL-2 abrogates the induction of autophagy [22]. On the other hand, high expression of BCL-2 inhibits not only autophagy but also apoptosis, thus influencing the cytotoxic response of ovarian cancer cells to chemotherapeutics [17, 21]. Thus, evaluating the level of expression of BECLIN 1 may not be sufficient to draw conclusions about the capacity of the cell to activate autophagy. We have analysed by western blotting the expression of BECLIN 1 and of BCL-2 in a small subset of carcinomas for which the frozen biopsy was available (representative cases are shown in Figure 4). In general, the expression of these proteins was inversely related. To seek for a functional relationship between the two proteins, we performed the immunostaining of BECLIN 1, BCL-2, and LC3 in two paradigmatic situations among the cases analysed by western blotting. In case 1, the expression of BCL-2 was quite high, which could account for inhibition of BECLIN 1 proautophagic activity, and in fact this tumour was negative for LC3 staining (Figure 5(a)). On the opposite, BCL-2 and BECLIN 1 were not detectable (by western blotting) in the tumour case 2, and in spite of this the tumour was intensely LC3 positive (Figure 5(b)), which possibly was associated with BECLIN 1-independent autophagy.

## 4. Discussion

Autophagy, a cell homeostatic process for the lysosome-driven degradation of aged, damaged, and redundant self-constituents, may either suppress or facilitate carcinogenesis [7, 23]. The heterozygous deletion of the autophagy gene* BECLIN 1* in transgenic mice predisposes to the development of spontaneous tumours, including ovarian cancers [11, 24]. Accordingly, the expression of the BECLIN 1 protein and also of the autophagosome protein LC3 was found much lower in malignant ovarian cancers compared to benign ovary epithelial tissues [16, 17]. In our series, we also have found that 18 out of 20 ovarian cancers of histologic grade III were negative or low expressing BECLIN 1. This is consistent with the view that defective autophagy might favour cancer progression. In this same line, a decreased level of BECLIN 1 expression, especially in conjunction with increased expression of BCL-xL, was correlated with poor prognosis in ovarian cancer bearing patients [17]. Here we have analysed the tissue expression of BECLIN 1 in a series of 61 cases of ovarian carcinomas of various histologic types. BECLIN 1 staining presented with either a cytoplasmic diffused pattern (regarded as negative) or a granular-like pattern (regarded as positive). The latter likely reflected the engagement of BECLIN 1 in the oligomeric interactome with PI3-kinase class III [12], which preludes to the initiation of autophagy [22]. Fourteen (of the 61) cancers examined showed positive for BECLIN 1 in a percentage of cells ranging from 20% to 90%. The expression of BECLIN 1 was not correlated with patient's age at the time of diagnosis, nor was it correlated with a particular histologic type. It is to be noted, however, that in our series some histologic types were underrepresented so that no conclusion could be drawn with regard to the association between autophagy and histotypes. On the other hand, being autophagy, an evolutionary conserved and ubiquitous process, it is conceivable that it is not restricted to a particular subtype of cancer. Setting the cut-off at 20% of positive cells (in terms of BECLIN 1 macroaggregates), a positive correlation was found between negative expression and high histologic grade. In general, the clinically indolent type I tumours were more frequently expressing BECLIN 1 at high level.

However, no statistically significant correlation was found between the positive expression and the pathological stage at diagnosis. Thus, while defective autophagy likely favours the emergence of highly malignant clones, other factors influence the general evolution of the disease in the patient.

Tumours negative for BECLIN 1 showed no correlation with prognosis (11 survivors and 9 DOD), whereas of the 41 patients bearing a BECLIN 1-positive tumours as many as 34 showed a favourable prognosis (24 CR and 10 PR). Seen from a different point, of the 32 patients that underwent CR as many as 24 were bearing a BECLIN 1-positive cancer and only 8 were bearing a BECLIN 1-negative cancer. These correlations were statistically significant. While our data seem to be consistent with the findings reported by Shen et al. [16] and Lin et al. [17], other authors have reported opposite findings. In one study [18], the expression of BECLIN 1 was inversely correlated with the histologic grade of differentiation of ovarian carcinomas and the high level of BECLIN 1 expression was associated with a lower relapse-free survival rate of the patients. High level of BECLIN 1 was also found associated with invasive endometrioid cancers and poor 5-year survival [19]. However, both in these studies BECLIN 1 was not an independent prognostic factor. In our series, we have indeed observed that seven patients bearing a cancer with >20% of BECLIN1-positive cells deceased within the follow-up period. Assuming that BECLIN 1 main function was to drive autophagy and that autophagy was playing a positive role in the response to chemotherapy treatments, we considered the possibility that failure in the chemotherapy response in those patients could arise from impaired (or insufficient) induction of autophagy in the tumour cells expressing BECLIN 1. To better detect autophagy active cells in the tumour, we stained the cells for LC3, an autophagosomal protein considered to be hallmark of ongoing autophagy [8]. In general, a high concordance between BECLIN 1 and LC3 positivity was observed in the large majority of the cases. In some cases, LC3 was negative in spite of the positivity for BECLIN 1. This fact was likely due to the concomitant high expression of BCL-2, which is known to nullify the autophagy function of BECLIN 1 [22], as was proven in at least some of the cases. We found that the BECLIN 1-positive cancers associated with the patients deceased during the study were indeed negative for vacuolar LC3 staining and highly expressing BCL-2. Though not statistically relevant because of the small number of cases, indirectly our finding agrees with that reported by Lin et al. [18], who showed that low expression of BECLIN 1 in combination with high expression of BCL-xL predicts a poor survival in ovarian cancer patients. Of note, also LC3 positivity significantly correlated with patient's overall survival at 5 years after diagnosis, thus supporting the contention that the patients bearing a tumour with a high proportion of autophagy-active cells had a better prognosis. In this regard, it is to be mentioned that, in clear cell ovarian cancer histotypes, but not in other examined histotypes, the high expression of LC3A was found to significantly correlate with hypoxia and poor prognosis [20]. We could not compare with this study, as in our series we had only 4 cases of clear cell carcinomas, 2 each either BECLIN 1 positive or BECLIN 1 negative.

It remains to be explained through which molecular pathway the ongoing autophagy in cancer cells could turn of benefit in the chemotherapeutic response so that the patient experiences a better prognosis. The two-hit model predicts a synergistic death effect of two proautophagic stimuli [25]. In fact, although autophagy is in principle a prosurvival pathway, it might also lead to cell death if dysregulated [12, 23, 26]. In particular, cells in which autophagy is basally upregulated may undergo apoptosis if subjected to an additional metabolic or genotoxic stress that hyperinduces autophagy [25]. We hypothesize that autophagy-active cancer cells may succumb in response to drugs that hyperstimulate autophagy. This is the rationale for the use of mTOR inhibitors in ongoing clinical trials for the treatment of ovarian cancers [7]. With relevance to our chemotherapy protocol, it has been reported that the transgenic overexpression of BECLIN 1 sensitizes cervical cancer cells to carboplatin and to paclitaxel by promoting apoptosis and autophagic cell death [27, 28]. BECLIN 1 and BCL-2 occupy a central role in the complex cross-talk between autophagy and apoptosis [29], and chemotherapeutic drugs could be more effective in those cells with an altered ratio between these two proteins. Consistent with our hypothesis, it was recently shown that the Src/Abl kinases inhibitor Dasatinib arrested the growth of ovarian cancer xenograft by inducing BECLIN 1-dependent autophagic cell death, and hyperstimulation of autophagy was associated with downregulation of BCL-2 expression [30]. This could also explain the poor survival reported in women bearing an ovarian cancer expressing low level of BECLIN 1 and high level of BCL-xL [17]. Besides, the high expression of BECLIN 1 could enhance the cytotoxic response to a chemotherapeutic drug in ovarian cancer cells also via an autophagy-independent mechanism [31]. Additionally, the hypothesis that tumour with intrinsic high level of basal autophagy may have a better prognosis even without chemotherapy cannot be excluded. Though we could not test directly this hypothesis, we note that of the 7 patients for whom chemotherapy was not deemed (because they were staged as pT1 and the tumour was of grade 1) 6 were bearing a BECLIN 1-positive tumour and underwent CR, whereas 1 was bearing a BECLIN 1-negative tumour and was DOD.

In conclusion, while on one hand the upregulation of basal autophagy associated with a higher ratio of BECLIN 1 versus BCL-2 proteins expression enables the cancer cells to overcome the metabolic stresses caused by the lack of oxygen and nutrients, it on the other hand also renders these cells more susceptible to chemotherapeutic drugs that overstimulate autophagy. Given the role of mitochondria in the apoptotic response to chemotherapeutics [32], we suspect that in the latter case apoptosis ensues because of the exaggerated mitophagy. Thus, to improve the chance to cure ovarian carcinomas, one should carefully consider whether to employ autophagy inhibitors or autophagy-enhancer drugs in the chemotherapy cocktail depending on the ratio of BECLIN 1 and BCL-2 expression and the actual level of autophagy in the cancer cells.

## Supplementary Material

Supplementary Table 1: Histologic and clinical characteristics of ovary carcinomas included in the study
Supplementary Fig 1: Expression of BECLIN 1 and clinical outcome

## Figures and Tables

**Table 1:** Distribution of BECLIN 1 positivity (in terms of H ≥ 40) among ovarian carcinoma histologic types.

BECLIN 1 positive	Yes	No	Number of cases
Histologic type I			
Serous (low grade)	8	0	8
Endometrioid (low grade)	8	0	8
Clear cell	2	2	4
Mucinous	2	1	3
Transitional (Brenner)	0	1	1

Histologic type II			
Serous (high grade)	11	8	19
Endometrioid (high grade)	3	2	5
Undifferentiated	6	5	11
Mixed mesodermal	1	1	2
Total	**41**	**20**	**61**

**(a):** 

Grade	I-II	III	Number of cases
BECLIN 1			
+	21	20	41
−	2	18	20
Total	**23**	**38**	**61**

Chi-square = 8.05

DF = 1

*P* = 0.0046

Fischer's test *P* = 0.002.

**(b):** 

Stage	I-II	III-IV	Number of cases
BECLIN 1			
**+**	24	17	41
−	10	10	20
Total	**34**	**27**	**61**

Chi-square = 0.13

DF = 1

*P* = 0.7

Fischer's test *P* = 0.59.

**(a):** 

Clinical outcome	Survivors	DOD	Number of cases
BECLIN 1			
+	34	7	41
−	11	9	20
Total	**45**	** 16**	** 61**

Chi-square = 4.07

DF = 1

*P* = 0.04

Fischer's test

*P* = 0.03.

**(b):** 

Clinical outcome	CR	PR	DOD	Number of cases
BECLIN 1				
**+**	24	10	7	41
−	8	3	9	20
Total	**32**	** 13**	**16**	** 61**

Chi-square = 5.4

DF = 2

*P* = 0.066.

**(a):** 

% LC3 positive	Survivors 5 y	DOD	Number of cases
<20%	3	6	9
≥20%	20	1	21
Total	**23**	**7**	**30**

Chi-square = 13.5

DF = 1

*P* = 0.0002.

**(b):** 

% LC3 positive	Survivors 5 y	DOD	Number of cases
<20%	7	11	18
≥20%	23	1	24
Total	**30**	**12**	**42**

Chi-square = 16.34

DF = 1

*P* = 0.0001.
