# Supplementary material for: Expression and Clinical Significance of the Autophagy Proteins BECLIN 1 and LC3 in Ovarian Cancer
Source: Biomed Res Int. 2014 Jul 17;2014:462658. doi: 10.1155/2014/462658 (PMC4127242; doi:10.1155/2014/462658)
Supplement: Supplementary file 1 — Supplementary Table 1: Histologic and clinical characteristics of ovary carcinomas included in the study Supplementary Fig 1: Expression of BECLIN 1 and clinical outcome [file 462658.f1.zip › SUPPLEMENTARY TABLE 1.docx]

**SUPPLEMENTARY TABLE 1 (ST1)**

| **A** | **Case Number** | **Age** | **pT** | **Histologic Type** | **G** | **Chem.** | **OUTCOME** | **BECN** | **LC3/ BECN+** | **LC3/ BECN-** |
| --- | --- | --- | --- | --- | --- | --- | --- | --- | --- | --- |
|  | **1** | 47 | 3c | Serous LG | 1 | Y | DOD | **+** | **+** |  |
|  | **2** | 49 | 1a | Serous LG | 1 | N | DOD | **+** | **-** |  |
|  | **3** | 55 | 1a | Serous LG | 2 | Y | PR | **+** |  |  |
|  | **4** | 82 | 2a | Serous LG | 2 | Y | CR | **+** | **+** |  |
|  | **5** | 66 | 2a | Serous LG | 2 | Y | CR | **+** | **+** |  |
|  | **6** | 33 | 3c | Serous LG | 2 | Y | DOD | **+** | **-** |  |
|  | **7** | 66 | 3c | Serous LG | 2 | Y | DOD | **+** | **-** |  |
|  | **8** | 58 | 3c | Serous LG | 2 | Y | CR | **+** | **+** |  |
|  | **9** | 56 | 1a | Endometrioid LG | 1 | N | CR | **+** | **+** |  |
|  | **10** | 59 | 1a | Endometrioid LG | 1 | N | CR | **+** | **+** |  |
|  | **11** | 65 | 1c | Endometrioid LG | 1 | N | CR | **+** | **+** |  |
|  | **12** | 40 | 3a | Endometrioid LG | 1 | Y | CR | **+** | **+** |  |
|  | **13** | 52 | 1a | Endometrioid LG | 2 | Y | CR | **+** | **+** |  |
|  | **14** | 77 | 1c | Endometrioid LG | 2 | Y | PR | **+** | **+** |  |
|  | **15** | 69 | 1c | Endometrioid LG | 2 | Y | CR | **+** | **+** |  |
|  | **16** | 68 | 3c | Endometrioid LG | 2 | Y | DOD | **+** | **-** |  |
|  | **17** | 62 | 1c | Clear Cell | 1 | N | CR | **+** | **-** |  |
|  | **18** | 81 | 1a | Clear Cell | 2 | Y | PR | **+** |  |  |
|  | **19** | 56 | 1c | Clear Cell | 3 | Y | CR | **-** |  | **+** |
|  | **20** | 75 | 1c | Clear Cell | 3 | Y | CR | **-** |  |  |
|  | **21** | 54 | 1a | Mucinous | 1 | N | CR | **+** | **+** |  |
|  | **22** | 60 | 1c | Mucinous | 2 | Y | CR | **-** |  |  |
|  | **23** | 47 | 3c | Mucinous | 3 | Y | CR | **+** | **-** |  |
|  | **24** | 84 | 1a | Brenner | 1 | N | DOD | **-** |  | **-** |

| **B** | **Case Number** | **Age** | **pT** | **Histologic Type** | **G** | **Chem.** | **OUTCOME** | **BECN** | **LC3 /BECN+** | **LC3 /BECN-** |
| --- | --- | --- | --- | --- | --- | --- | --- | --- | --- | --- |
|  | **25** | 74 | 2a | Serous HG | 2 | Y | PR | **+** |  |  |
|  | **26** | 68 | 1a | Serous HG | 3 | Y | CR | **+** | **+** |  |
|  | **27** | 44 | 1c | Serous HG | 3 | Y | PR | **+** |  |  |
|  | **28** | 82 | 1c | Serous HG | 3 | Y | CR | **+** | **+** |  |
|  | **29** | 53 | 1c | Serous HG | 3 | Y | PR | **+** |  |  |
|  | **30** | 66 | 1c | Serous HG | 3 | Y | CR | **+** | **+** |  |
|  | **31** | 29 | 2a | Serous HG | 3 | Y | CR | **-** |  | **-** |
|  | **32** | 65 | 2a | Serous HG | 3 | Y | CR | **-** |  | **-** |
|  | **33** | 57 | 2c | Serous HG | 3 | Y | DOD | **-** |  | **-** |
|  | **34** | 64 | 2c | Serous HG | 3 | Y | CR | **+** | **+** |  |
|  | **35** | 70 | 3c | Serous HG | 3 | Y | DOD | **+** | **-** |  |
|  | **36** | 80 | 3b | Serous HG | 3 | Y | PR | **-** |  |  |
|  | **37** | 80 | 3c | Serous HG | 3 | Y | PR | **-** |  |  |
|  | **38** | 60 | 3c | Serous HG | 3 | Y | DOD | **-** |  | **-** |
|  | **39** | 51 | 3c | Serous HG | 3 | Y | DOD | **+** | **-** |  |
|  | **40** | 68 | 3c | Serous HG | 3 | Y | DOD | **-** |  |  |
|  | **41** | 71 | 3c | Serous HG | 3 | Y | PR | **+** |  |  |
|  | **42** | 60 | 3c | Serous HG | 3 | Y | CR | **+** | **+** |  |
|  | **43** | 57 | 4a | Serous HG | 3 | Y | CR | **-** |  | **+** |
|  | **44** | 38 | 1c | Endometrioid HG | 3 | Y | CR | **+** | **+** |  |
|  | **45** | 77 | 3b | Endometrioid HG | 3 | Y | DOD | **-** |  |  |
|  | **46** | 52 | 3c | Endometrioid HG | 3 | Y | CR | **+** |  | **-** |
|  | **47** | 81 | 3c | Endometrioid HG | 3 | Y | DOD | **-** |  | **-** |
|  | **48** | 60 | 3c | Endometrioid HG | 3 | Y | PR | **+** |  |  |
|  | **49** | 53 | 3c | Undifferentiated | 1 | Y | PR | **+** |  |  |
|  | **50** | 73 | 1c | Undifferentiated | 1 | N | DOD | **-** |  | **-** |
|  | **51** | 57 | 2a | Undifferentiated | 3 | Y | CR | **+** | **-** |  |
|  | **52** | 40 | 2a | Undifferentiated | 3 | Y | DOD | **-** |  |  |
|  | **53** | 73 | 2c | Undifferentiated | 3 | Y | PR | **-** |  | **-** |
|  | **54** | 56 | 3a | Undifferentiated | 3 | Y | CR | **+** | **+** |  |
|  | **55** | 45 | 3b | Undifferentiated | 3 | Y | DOD | **-** |  |  |
|  | **56** | 37 | 3c | Undifferentiated | 3 | Y | CR | **+** |  |  |
|  | **57** | 59 | 3c | Undifferentiated | 3 | Y | CR | **-** |  |  |
|  | **58** | 40 | 3c | Undifferentiated | 3 | Y | CR | **+** | **+** |  |
|  | **59** | 40 | 3c | Undifferentiated | 3 | Y | PR | **+** |  |  |
|  | **60** | 57 | 1c | Mixed Mesodermal | 3 | Y | CR | **-** |  | **+** |
|  | **61** | 53 | 1c | Mixed Mesodermal | 3 | Y | CR | **+** | **+** |  |
