# Supplementary figures and images for: Expression and Clinical Significance of the Autophagy Proteins BECLIN 1 and LC3 in Ovarian Cancer
Source: Biomed Res Int. 2014 Jul 17;2014:462658. doi: 10.1155/2014/462658 (PMC4127242; doi:10.1155/2014/462658)

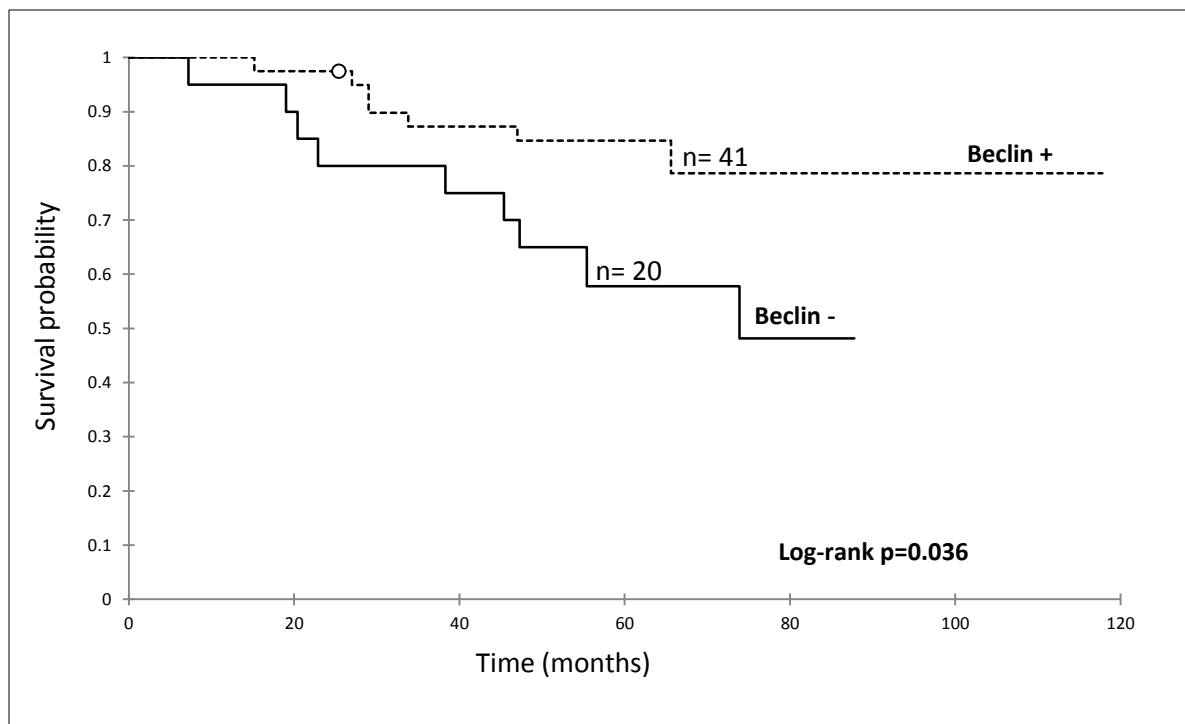

Supplement: Supplementary file 1 — Supplementary Table 1: Histologic and clinical characteristics of ovary carcinomas included in the study Supplementary Fig 1: Expression of BECLIN 1 and clinical outcome [file 462658.f1.zip › mat.462658.v2.pdf]
