# Supplementary material for: Expression and Clinical Significance of the Autophagy Proteins BECLIN 1 and LC3 in Ovarian Cancer
Source: Biomed Res Int. 2014 Jul 17;2014:462658. doi: 10.1155/2014/462658 (PMC4127242; doi:10.1155/2014/462658)
Supplement: Supplementary file 1 — Supplementary Table 1: Histologic and clinical characteristics of ovary carcinomas included in the study Supplementary Fig 1: Expression of BECLIN 1 and clinical outcome [file 462658.f1.zip › Description.doc]

**LEGEND TO SUPPLEMENTARY TABLE 1 (ST1)**

***Histologic and clinical characteristics of ovary carcinomas included in the study***

The complete database of the cases studied is shown in ST1. The principal information on the patient (age), the tumour (histologic type and grading), and the clinical data (staging, treatment, outcome) are reported. The tumours have been grouped as type I (upper part, A) and type II (lower part, B). Patients who underwent adjuvant chemotherapy with Carboplatin/Paclitaxel are indicated with Y (Yes). Clinical outcomes were classified as Complete Remission CR, or alternatively as NED: Not Evidence of Disease; i.e., disappearance of any evidence of disease during the follow-up or for at least four years), partial remission (PR, ≥ 50% decrease of tumour lesions for at least 24 months) and DOD (Dead Of Disease). The positivity for the expression of BECLIN 1 and of LC3 is reported.

Abbreviations: G, histologic grading; LG, low grade; HG, high grade; BECN, BECLIN 1, LC3, Light Chain 3.

**LEGEND TO SUPPLEMENTARY FIG. 1 (SF1)**

***Expression of BECLIN 1 and clinical outcome***

The Kaplan–Meier method was applied to assess the potential of BECLIN 1 expression to convey prognostic information. Overall survival was calculated from the date of diagnosis until the date of last follow-up or patient’s death. Surviving patients were censored at the date of last contact. The Log-rank test was applied to calculate the statistical significance of differences between the survival curves. Log-rank test indicated that the association of high expression of BECLIN 1 in the tumour with a good prognosis was statistically significant.
